# Identification of FDA-approved drugs targeting the Farnesoid X Receptor

**DOI:** 10.1038/s41598-019-38668-7

**Published:** 2019-02-18

**Authors:** Sandra M. W. van de Wiel, Ingrid T. G. W. Bijsmans, Saskia W. C. van Mil, Stan F. J. van de Graaf

**Affiliations:** 1 Tytgat Institute for Liver and Intestinal Research, Amsterdam Gastroenterology and Metabolism, Amsterdam UMC, University of Amsterdam, Amsterdam, The Netherlands; 2 Department of Gastroenterology and Hepatology, Amsterdam Gastroenterology and Metabolism, Amsterdam UMC, University of Amsterdam, Amsterdam, The Netherlands; 3 Center for Molecular Medicine, UMC Utrecht, Utrecht University, Utrecht, The Netherlands

## Abstract

The farnesoid X receptor (FXR) belongs to the nuclear receptor family and is activated by bile acids. Multiple, chemically rather diverse, FXR agonists have been developed and several of these compounds are currently tested in clinical trials for NAFLD and cholestasis. Here, we investigated possible FXR-agonism or antagonism of existing FDA/EMA-approved drugs. By using our recently developed FRET-sensor, containing the ligand binding domain of FXR (FXR-LBD), 1280 FDA-approved drugs were screened for their ability to activate FXR in living cells using flow cytometry. Fifteen compounds induced the sensor for more than twenty percent above background. Real-time confocal microscopy confirmed that avermectin B1a, gliquidone, nicardipine, bepridil and triclosan activated the FRET sensor within two minutes. These compounds, including fluticasone, increased mRNA expression of FXR target genes *OSTα* and *OSTβ* in Huh7 cells, and in most cases also of *MRP2*, *SHP* and *FGF19*. Finally, avermectin B1a, gliquidone, nicardipine and bepridil significantly increased IBABP promoter activity in a luciferase reporter assay in a dose-dependent manner. In conclusion, six FDA/EMA-approved drugs currently used in the clinical practice exhibit moderate agonistic FXR activity. This may on the one hand explain (undesired) side-effects, but on the other hand may form an opportunity for polypharmacology.

## Introduction

Bile acids are responsible for effective absorption of fats and fat-soluble vitamins, facilitate digestion and are important regulators of cholesterol, triglyceride homeostasis and inflammation^1–3^. Several of these metabolic actions of bile acids involve the activation of the nuclear farnesoid X receptor (FXR). FXR is used as a target for new drug therapies against metabolic dysregulation associated with obesity, including type 2 diabetes, non-alcoholic fatty liver disease (NAFLD), and atherosclerosis as well as for the cholestatic liver disease primary biliary cholangitis^4–7^. FXR is primarily expressed in liver, kidney, and intestine and is crucial for maintaining bile acid homeostasis^8^. Through heterodimerization with the retinoid X receptor (RXRs), FXR regulates transcription of target genes by binding to FXR response elements (FXREs)^9^. FXR regulates bile acid synthesis in the liver via inducing transcription of small heterodimer partner (SHP), which in turn inhibits LRH-1-mediated expression of cholesterol-7alpha-hydroxylase (CYP7A1), the key enzyme of bile acid synthesis^10^. In the intestine, FXR is activated after reabsorption of bile acids in the ileum, promoting amongst others transcription of fibroblast growth factor 15/19 (FGF15, in rodents, FGF19 in humans)^11^. After entering the portal circulation, FGF15/19 activates FGF receptor 4 (FGFR4) in the liver to suppress bile acid synthesis, thereby mediating important cross-talk between liver and intestine^12^.

Furthermore, FXR regulates the expression of bile acid transporters in liver, kidney and intestine. In general, bile acid induced activation of FXR enhances bile acid excretion from hepatocytes (BSEP, OSTα-OSTβ) and enterocytes (OSTα-OSTβ)^13,14^. This mechanism is crucial for bile acid homeostasis and protects against bile acid toxicity^15^.

Pharmacological activation of FXR with specific agonists has shown promising results in several patient studies. Activation of FXR by obeticholic acid (OCA) increased insulin sensitivity and reduced markers of liver inflammation and fibrosis in patients with type 2 diabetes and NAFLD^16^. Furthermore, OCA treatment in PBC patients significantly decreased bilirubin and ALP serum levels^17^. However, it also increased the risk for pruritus.

A number of different screens have been developed to identify FXR ligands. Most screens published to date are based on the AlphaScreen technology, which is a bead-based chemistry method to identify ligands that induce the recruitment of a coactivator (SCR1-2) to the ligand binding domain of the nuclear receptor FXR^18^. In a recent study, we screened for FDA/EMA-approved inhibitors of the organic solute transporter alpha-beta using a FRET bile acid sensor^19^. FRET-based sensors enable the quantitative analysis of metabolite dynamics on single-cell level in real time, and have been used successfully for many biomedical applications^20^. The FRET sensor used in this study (nucleoBAS, a nuclear Bile Acid Sensor) consists of an FXR ligand binding domain (LBD), an LXXLL motif, a nuclear localization sequence and an α-helical motif present in coactivators required for the ligand-dependent binding of coactivators to nuclear receptors. Weak, hydrophobic interactions between the fluorophores citrine and cerulean were stimulated by inserting V224L mutations, substantially increasing the dynamic range of the sensor^21^. Binding of compounds to the FXR LBD leads to recruitment of the LXXLL motif, bringing the fluorophores to close vicinity, and a concomitant change in FRET signal. In that study, we aimed to find inhibitors of OSTα-OSTβ, a bile acid exporter protein. However, also in cells lacking OSTα-OSTβ, 15 compounds activated the nucleoBAS sensor and we hypothesize that these hits are (weak) FXR ligands which activate the sensor directly.

In order to test this hypothesis, we characterized the FXR activating potential of drugs that are currently used in clinical practice. Fifteen drugs were considered positive for FXR activation (>20% above background levels) in the screen, and at least 6 drugs currently widely used in clinical practice bind and activate FXR. This implies that these drugs affect multiple target proteins, which is a common phenomenon^22^. Polypharmacology, or drug activity on multiple targets, forms the underlying mechanism of many side-effects and the subsequent drug withdrawal from the market^23^, but may also open up the possibility for modulating multiple targets with one drug^24^.

## Results

### The nucleoBAS sensor is activated by several prescription drugs

**Figure 1:**
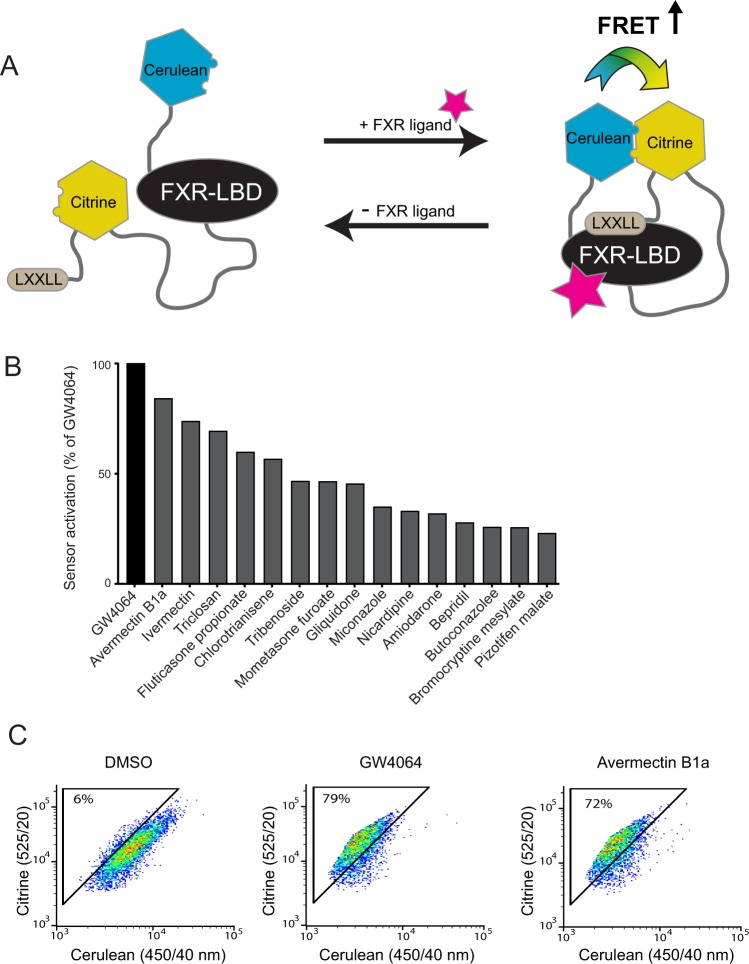
High-throughput screening of FXR ligands using FRET-based flow cytometry. (**A**) working model of the FRET sensor. (**B**) Mean values of the fifteen strongest hits that increased the FRET+ cells for at least 20%. (**C**) Representative FACS-plots showing the amount of FRET+ cells after 30 minute treatment with negative control (DMSO), positive control (GW4064) or 10 μM of the top hit avermectin B1a. Numbers indicate the percentages of cells within the FRET gate.

To explore whether general prescription drugs bind and activate FXR, FRET efficiency was measured in nucleoBAS-transfected U2OS cells that lack expression of bile acid transporters. A robust change in emission ratio of the FRET-bile acid sensor is observed upon ligand binding (Fig. 1A). Out of 1280 compounds present in the Prestwick library, fifteen drugs induced the nucleoBAS sensor more than 20% relative to DMSO treated cells (0%) and GW4064 (100%) (Fig. 1B). Flow cytometry plots of cerulean and citrine intensity of controls and of the top hit Avermectin B1a is shown in Fig. 1C. More FACS plots are shown in Supplementary Fig. 1. There were no apparent structure similarities among the top 10 hits and other known FXR ligands (Table 1).

**Table 1:** Hits identified from the FRET-based screen.

Chemical name	Therapeutic effect	Structure	Structure formula	% FXR activation relative to GW4064
Avermectin B1a (Abamectin)	Antihelmintic		C48H72O14	84%
Amiodarone HCl	Antianginal		C25H30ClI2NO3	32%
Bepridil HCl	Antianginal		C24H35ClN2O	28%
Fluticasone proprionate	Anti-inflammatory		C25H31F3O5S	60%
Fulvestrant	Antineoplastic		C32H47F5O3S	17%
Gliquidone	Antidiabetic		C27H33N3O6S	45%
Ivermectin	Antihelmintic		C48H74O14	74%
Nicardipine HCl	Antianginal		C26H30ClN3O6	33%
Triclosan	Antibacterial		C12H7Cl3O2	69%

### Validation of putative FXR-activating compounds using luciferase reporter assays

**Figure 2:**
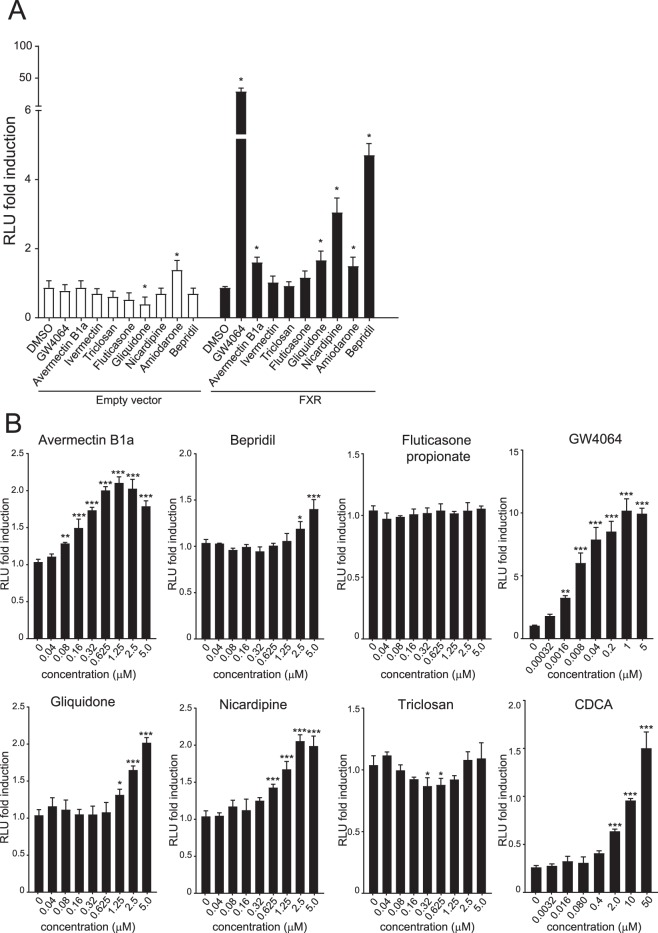
Luciferase reporter assay with IBABP promoter. HEK293T cells were cotransfected with FXR (pcDNA3.1-FXRα2), RXR (pcDNA3.1-RXRα) and the IBABP promoter construct (pGL3-IBABP/FABP6). (**A**) Cells were treated with 10 μM compound (or 5 µM abamectin and GW4064) or DMSO for 24 hours and harvested for Firefly and Renilla luciferase reporter assays. (**B**) RLU fold induction after 24 hours treatment with a dose range of compounds. The x-axis depicts the concentration in micromolar (µM). Data are presented as mean ± SD expressed relative to DMSO treated cells. This experiment (n = 4) was performed in duplicate. *P < 0.05; **P < 0.01; ***P < 0.001. (One-way ANOVA; post hoc: Dunnett’s multiple comparison).

Next, we used a luciferase reporter assay to determine whether the identified compounds could activate transcription from the IBABP promoter, a well-established FXR target gene^25,26^. HEK293T cells were transiently transfected with an IBABP reporter construct (a luciferase gene fused to the IBABP gene promoter) in combination with RXR and FXR expression plasmids. Reporter activity was induced upon treatment with avermectin B1a, gliquidone, nicardipine, amiodarone and bepridil compared to DMSO treated cells (Fig. 2A). With the exception of amiodarone, none of these compounds activated the reporter in the absence of transfected FXR, indicating that the observed effects are FXR-dependent. Moreover, avermectin B1a, bepridil, gliquidone and nicardipine induced the IBABP reporter activity in a dose-dependent manner (Fig. 2B), while this was not observed in cells without FXR (data not shown). Next, we addressed two questions: (1) Do these hits directly bind and activate FXR? and (2) is there an effect on FXR target gene expression?

### Monitoring of FXR activation in living cells

**Figure 3:**
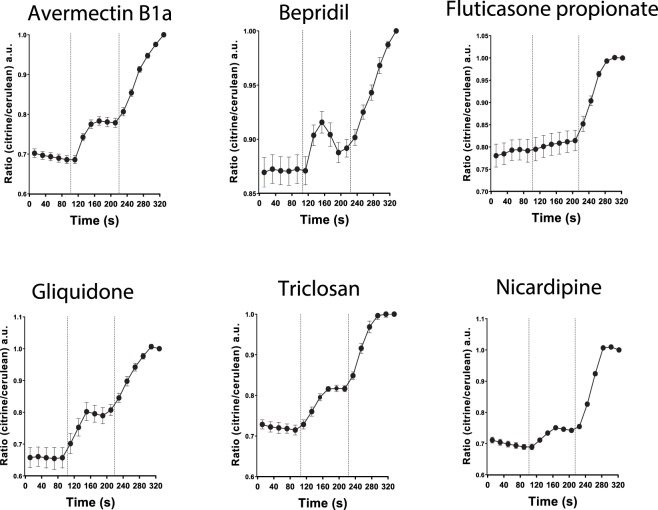
FRET imaging of compound induced conformational change of FXR. Ratio of measured fluorescence intensity of fluorophores cerulean (donor) and citrine (acceptor) plotted in time during administration of 10 μM compound (t = 110 s) and 5 μM GW4064 (t = 230 s), n = 6.

The nucleoBAS sensor offers the possibility to measure robust changes in emission ratios with a high temporal resolution in living cells upon substrate binding to the sensor. To distinguish between slow (30 minutes) and rapid (within 2 minutes) FXR-ligand binding to the sensor, the conformational change of the nucleoBAS sensor upon binding was visualized using confocal microscopy. Emission of citrine and cerulean was measured to determine the amount of FRET signal in region of interest (ROIs) of individual cells. Stimulation of sensor expressing U2OS cells with 10 μM avermectin B1a, bepridil, gliquidone, triclosan and nicardipine (administered at t = 100 seconds) evoked a moderate increase of fluorescence intensity of the acceptor citrine and a decrease of the donor cerulean (Fig. 3). Fluticasone did not show any effect. At t = 230 seconds, cells were treated with FXR agonist GW4064 to determine maximum FRET intensity. These data suggest direct binding of avermectin B1a, bepridil, gliquidone, triclosan and nicardipine to the FXR ligand binding domain of the sensor, leading to the recruitment of the coactivator peptide LXXLL. Fluticasone did not affect the sensor within this short time period, but may activate FXR in a different way.

### Putative FXR-activating compounds induce endogenous mRNA expression of FXR target genes

To determine whether these putative FXR ligands are able to activate mRNA expression of endogenously expressed FXR target genes, human hepatocarcinoma cells (Huh7) were treated with DMSO, 10 μM CDCA, 10 μM GW4064, 5 μM avermectin B1a or 10 μM bepridil, fluticasone, gliquidone, triclosan or nicardipine for 6 hours. Subsequently, mRNA expression levels of FXR target genes were examined. All compounds except for fluticasone induced expression of FXR target gene *OSTα* (avermectin B1a: 4 fold, bepridil: 3.5 fold, gliquidone: 3.5 fold, triclosan: 2.5 fold and nicardipine: 4 fold). *OSTβ* mRNA expression was induced more than 2.5 fold upon treatment with avermectin B1a, gliquidone, triclosan and nicardipine (Supplementary Fig. 2).

**Figure 4:**
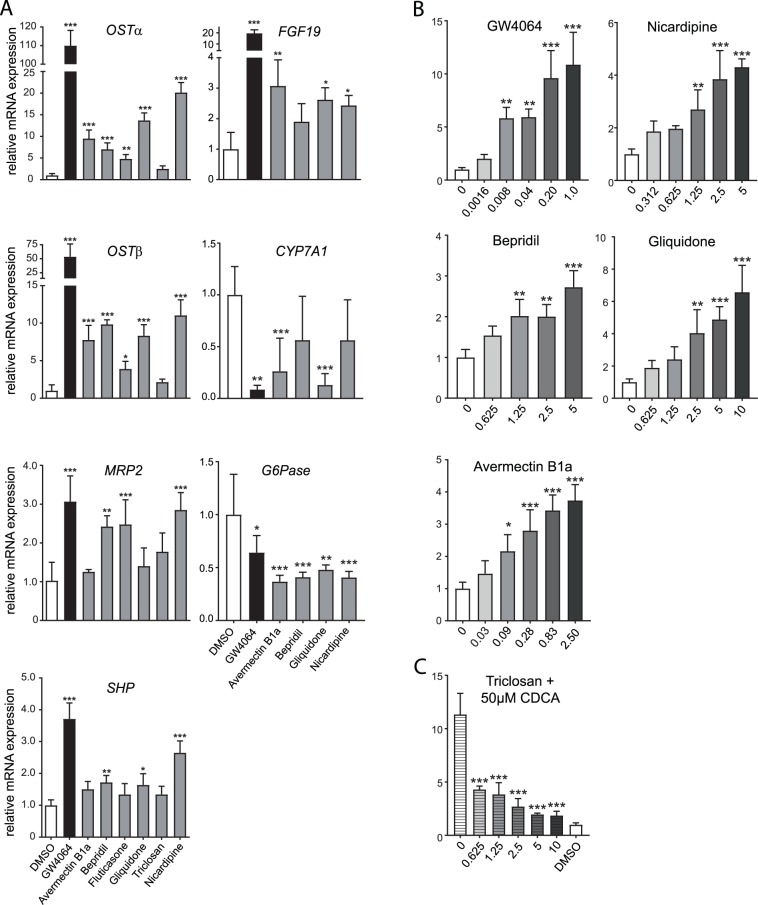
Compounds increase mRNA expression of FXR target genes. (**A**) Huh7 cells were treated for 72 hours with either DMSO, 5 μM GW4064, 5 μM abamectin B1a or 10 μM of compound and were analysed for differences in gene expression of FXR target genes *OSTα*, *OSTβ*, *MRP2*, *SHP*, *FGF19*, *CYP7A1 and G6Pase*. This experiment (n = 4) was performed in triplicate. (**B**) *OSTβ* mRNA expression levels after 24 hours treatment with a dose range of compounds or GW4064. The x-axis depicts the concentration in micromolar (µM). (**C**) *OSTβ* mRNA expression levels after 24 hours treatment with a dose range of triclosan combined with 50 µM CDCA. The x-axis depicts the concentration in micromolar (µM). Data are given as mean ± SD. Significance is determined relative to DMSO controls, n = 4. *p < 0.05; **p < 0.01; ***p < 0.001. (One-way ANOVA; post hoc: Dunnett’s multiple comparison).

Long-term treatment (72 hours) induced FXR target gene expression even more, and led up to 20-fold inductions (Fig. 4A). Incubation of Huh7 cells with all tested compounds, except for triclosan, elevated *OSTα* and *OSTβ* mRNA expression levels significantly, suggesting that they are FXR agonists. *MRP2* mRNA gene expression was upregulated after treatment with bepridil, fluticasone and nicardipine. Expression levels of *SHP* were upregulated after treatment with bepridil, gliquidone and nicardipine. Avermectin B1a, gliquidone and nicardipine were the only compounds that upregulated *FGF19* gene expression and avermectin B1a and gliquidone also reduced *CYP7A1* expression. Gene expression of *G6Pa*se, an important enzyme in the control of glucose homeostasis that is negatively regulated by FXR, was strongly reduced after treatment with avermectin B1a, bepridil, gliquidone and nicardipine. This implies that five out of six compounds are found to be FXR agonist, although with differences in potency or affinity. Triclosan was the only drug not inducing FXR target gene expression. Different conformational changes of the receptor by these ligands may allow for preferential binding to some FXR response elements but not others. In addition, we investigated whether a dose-dependent effect could also be observed in FXR target gene expression. Indeed, avermectin B1a, bepridil, gliquidone and nicardipine enhanced *OSTβ* mRNA expression in a dose-dependent manner (Fig. 4B). Furthermore, we investigated the ability of triclosan to exert antagonistic activity on FXR in cells co-incubated with 50 µM CDCA. Triclosan is either a partial antagonist or a very weak agonist competing with CDCA in a dose-dependent manner (Fig. 4C).

## Discussion

In this study, we explored whether FDA/EMA-approved drugs designed for other targets also activate FXR. Considering the small size of the library (1280 compounds), a relatively large number of compounds that had the ability to activate FXR was identified in the initial screen. Since several nuclear receptors are known for their flexible ligand-binding domain, this is not entirely unexpected^27,28^. Here, we provide data of at least six drugs currently used in clinic practice that bind FXR and induce mRNA expression levels of FXR target genes.

There is no obvious resemblance between these top hits compounds or with endogenous bile acids based on their structure. However, also amongst FXR activating compounds currently tested in clinical trials, there are also non-steroidal compounds that have no similar structures to bile acids or GW4064, indicating that the FXR ligand binding domain is able to bind various structurally different compounds^29^. Some hits identified in this screen have already been described as FXR modulating compounds, and demonstrate the robustness of this screen. For instance, both ivermectin and avermectin B1a were recently discovered as weak and partial agonists of FXR^30,31^. Furthermore, nicardipine has been previously described as an FXR agonist and also amiodarone has been associated with the regulation of glucose and lipid metabolism via FXR before^32^. Furthermore, mometasone furoate is closely related to fluticasone propionate, one of our hits, and both induce FXR while actually being developed as glucocorticoid receptor agonists^33,34^. Surprisingly, fulvestrant is one of the hits found in our screen with 17% increased FRET efficiency compared to DMSO, however, fulvestrant has already been described as an antagonist of FXR^35^. This raises the possibility that perhaps fulvestrant is a weak agonist and therefore acts primarily as an antagonist of FXR, for instance by preventing bile acids to bind. Nevertheless, not all hits from this screen have been discovered before. For instance, fluticasone propionate, triclosan and bepridil have not yet been described as potential FXR ligands. This is probably due to the design, execution or readout of the screens that can differ greatly. In contrast to other screens, the nucleoBAS sensor presents the opportunity to study the ligand-sensor interaction in high temporal resolution in living cells^36^. Nonetheless, no screen is best for all possible applications, as each has its merits and challenges. Therefore, the existence of several methods can be regarded as an advantage.

Fluticasone propionate activated the sensor within 30 minutes in the primary screen, but did not result in increased FRET values immediately after fluticasone propionate administration within the three minute confocal experiment. There may be several explanations for this discrepancy. The delay in FRET activity may be due to inefficient transport of the drug fluticasone propionate into U2OS cells. Or that fluticasone propionate needs to be metabolized before it can activate FXR. This would not be surprising if one considers that some cell lines have lower expression of P450 genes than others, which would prevent metabolization of certain compounds.

A relevant point to consider is the likelihood of these drugs reaching FXR expressing tissues, when given through the prescribed administration route. The probability that oral drugs (gliquidone, amiodarone, nicardipine, bepridil and triclosan) reach FXR in enterocytes is certainly high. However, the prospect that topical drugs like ivermectin and avermectin B1a come in contact with FXR is considerably lower, since the skin is a barrier to many compounds. Especially when taken into account the 500 dalton rule for skin penetration, which predicts that both ivermectin and avermectinB1A (>800 dalton) cannot pass the corneal layer^37^. Nasal drugs like fluticasone propionate often include rapid and high systemic activation and could, besides the gut, easily reach other FXR expressing tissues such as the liver, adrenal gland and kidney^26,38^.

A subsequent question remains whether some drug effects can now be assigned to FXR activation. Fluticasone propionate and mometasone furoate are both anti-inflammatory drugs, which can be linked to FXR activation since FXR is known for its anti-inflammatory effect^39^. Of particular interest is the drug gliquidone, an oral drug used to treat patients suffering from type 2 diabetes mellitus^40^. This drug is targeting an ATP-dependent K+ channel blocker leading to insulin release and reduced plasma glucose levels^41^. Likewise, findings of several studies support a role for FXR in glucose homeostasis, that could work in synergy to reduce glucose levels even further^42^. Another interesting drug to discuss is triclosan, an antibacterial agent found in many consumer products, for instance first aid products, toothpaste, mouthwash and soap. Many of these products are used regularly, and as a consequence, people may be exposed to FXR activators regularly. Furthermore, triclosan is rapidly absorbed through skin and can enter the bloodstream for systemic circulation. Interestingly, a recent study in which mice were briefly exposed to relatively low triclosan concentrations showed adverse health effects in mice with colitis, as demonstrated by increased gut inflammation and enhanced colon cancer cell growth^43^. Moreover, triclosan treated mice showed accelerated hepatocellular carcinoma development^44^. Similarly, FXR deficiency also leads to increased inflammation and enhances tumour growth in gut and liver^45,46^. Triclosan was one of the strongest hits in the primary screen, but did not show an effect on IBABP promoter activity nor did it increase mRNA expression of FXR target genes after 72 hours. Based on the result that CDCA induced *OSTβ* gene expression is dose dependently decreased by triclosan, we conclude that triclosan either is a very weak agonist, or like fulvestrant, works as a (partial) antagonist and possibly competes with CDCA for binding of FXR, thereby inhibiting FXR. This may at least cause some of the negative effects observed after long-term exposure to triclosan. However, further research has to reveal whether there is a connection between these drugs and FXR.

In conclusion, at least six compounds from a library of 1280 FDA/EMA-approved drugs could activate FXR *in vitro* and enhance transcription of its target genes. Screens like the one described in this paper could provide insight in multiple drug targeting and will give us a more complete understanding of the requirements of FXR ligand binding.

## Material and Methods

### Reagents

The Prestwick chemical library® (1280 FDA/EMA approved compounds, 10 µM in 96-well plates) was purchased from Prestwick chemicals. Avermectin B1a, bepridil, fluticasone proprionate, gliquidone, triclosan and nicardipine were purchased from Sigma-Aldrich.

### Cell culture

All cells were cultured in DMEM medium (high glucose) supplemented with 10% fetal bovine serum (FBS), 1% pen/strep and 1% L-glutamine. NucleoBAS (Bile Acid Sensor localized in the nucleus)^21^ expressing U2OS cells were engineered by transfecting cells using polyethylenimine (PEI) as earlier described by Van de Wiel *et al*., JoVE, 2016^36^. Stable cell lines were generated by colony picking using cloning rings over well separated colonies as described before^36^. All cells were cultured at 5% CO_2_ at 37 °C.

### Fluorescence activated cell sorting (FACS) and confocal microscopy based FRET-Bile Acid Sensor analyses

Two days before the experiments, wild-type and transfected (NucleoBAS) U2OS cells were cultured in 5% charcoal treated fetal bovine serum, to prevent bile acid overload of the sensor. The adherent cell layer was trypsinized by 5 mM EDTA to create a suspension of single cells for FACS analysis. Cells were harvested by centrifugation and the resulting pellet was suspended in FACS uptake buffer (0.3 mM EDTA, 0.5% BSA, 0.01% NaN_3_ and 10 mM D-glucose), plated in 96-wells and subsequently incubated with 10 µM of one of 1280 compounds of the Prestwick chemical library for 30 minutes while shaking. Citrine and Cerulean were excited by a violet 405 nm laser. Fluorescence was collected in the 450/40 range for cerulean and 525/25 range for citrine. The ability of compounds to activate FXR can be easily measured using the sensor by analysing the ratio of citrine/cerulean. For confocal microscopy-based analyses of the FRET sensor, cells stably expressing a FRET-Bile Acid Sensor in the nucleus were seeded in a sterile 8-well coverslip bottomed chamber slide. Live cell fluorescent imaging was performed using a Leica SP8X-SMD confocal microscope with a fully enclosed 37 °C incubation cabinet, as previously described^36^.

### Luciferase reporter assays

HEK293T cells were grown in 96-multiwell plates and co-transfected with empty pGL3 or pGL3-IBABP reporter, tK-Renilla and either empty pcDNA or pcDNA-hFXRα2 together with pcDNA-RXRα using the standard calcium phosphate method, as described elsewhere^47^. After 24 h, cells were incubated with vehicle (DMSO), 10 μM GW4064 or 10 μM compound for 24 h. Cells were lysed and Firefly and Renilla luciferase activity were measured according to manufacturer’s instructions (Promega Dual-Luciferase Reporter Assay System, Promega, Madison, Wisconsin, USA) with the Centro LB 960 luminometer (Berthold Technologies, Vilvoorde, Belgium).

### Analysis of gene expression using quantitative real-time Polymerase Chain Reaction

Huh7 cells were plated in 12-wells. When attached, cells were treated with 10 μM compounds or DMSO in complete DMEM for 6, 24 or 72 hours. Total RNA was extracted from cells using TRIzol reagent (Invitrogen, Bleiswijk, The Netherlands) according to the manufacturer’s instructions. cDNA synthesis was initiated from 1 µg of DNAse-treated RNA using oligo dT primers and Superscript II reverse transcriptase (Invitrogen).

Quantitative RT-PCR was performed using SensiFAST SYBR No-ROX kit (Bioloine, Londen, UK) in a Roche lightcycler 480 II. Expression levels of all samples were normalized to the geometrical mean of housekeeping genes. Raw data was converted using LC480 conversion software and samples were individually checked for their baseline and amplification efficiency using LinRegPCR software. Primer oligonucleotide sequences are all listed in Supplementary Table 1.

### Statistical analysis

Data are presented as the mean ± standard deviation. The results obtained were statistically analysed using one-way ANOVA; post hoc: Dunnett’s multiple comparison or Student’s t-test, two-tailed. Results were considered statistically significant at a P-value of <0.05.

## Data Availability

The datasets generated during and/or analysed during the current study are available from the corresponding author on reasonable request.

## Supplementary information

Supplementary Information
