# Supplementary material for: Identification of FDA-approved drugs targeting the Farnesoid X Receptor
Source: Sci Rep. 2019 Feb 18;9:2193. doi: 10.1038/s41598-019-38668-7 (PMC6379390; doi:10.1038/s41598-019-38668-7)
Supplement: Supplementary file 1 — Supplementary Information [file 41598_2019_38668_MOESM1_ESM.pdf]

Supplementary Data

Identification of FDA-approved drugs targeting the Farnesoid X receptor

Sandra M.W. van de Wiel[1,2], Ingrid T.G.W. Bijsmans[3], Saskia W.C. van Mil[1,3] and Stan F.J. van de Graaf[1,2]

[1]Tytgat Institute for Liver and Intestinal Research & [2]Department of Gastroenterology and Hepatology, Amsterdam Gastroenterology and Metabolism, Amsterdam UMC, University of Amsterdam, Amsterdam, the Netherlands. [3]Center for Molecular Medicine, UMC Utrecht, Utrecht University, Utrecht, The Netherlands.

Supplement Figure 1

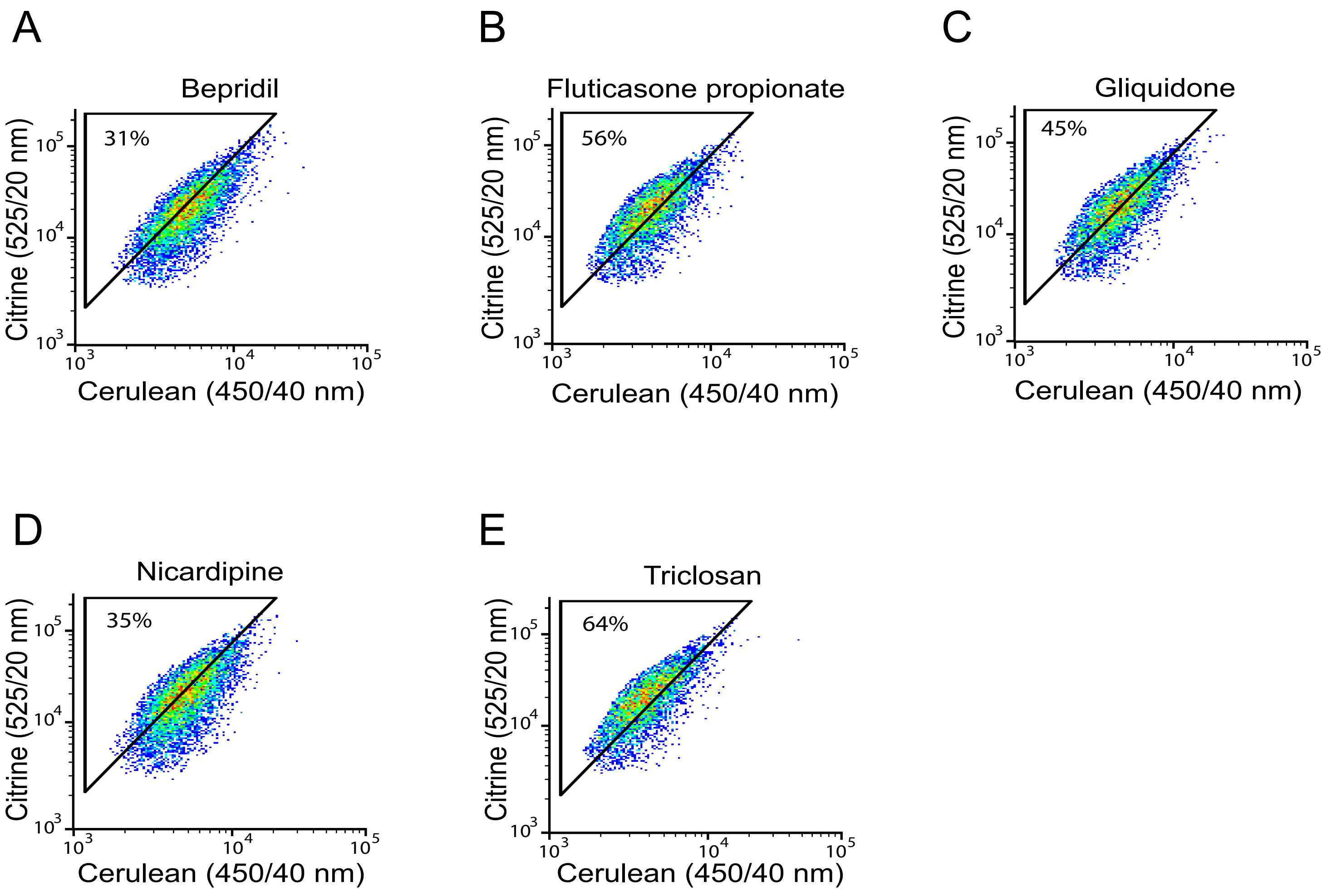

Supplement Figure 2

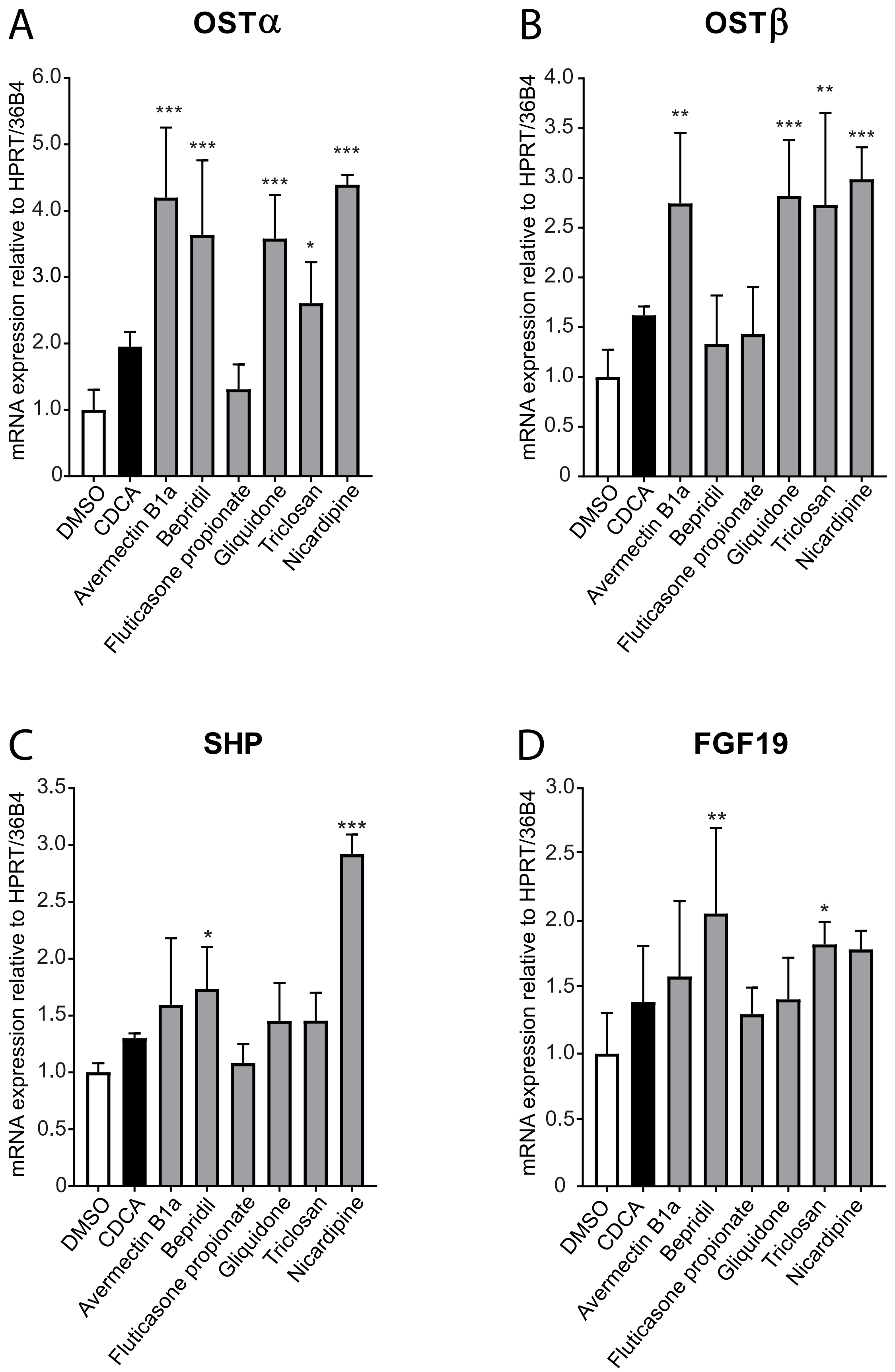

Supplementary figure 1: Representative FACS-plots of five hits showing the amount of FRET+ cells after 30 minute treatment with negative control (DMSO), positive control (GW4064) or 10 μM of compound dissolved in DMSO. Numbers indicate the percentages of cells within the FRET gate.

Supplementary figure 2: Compounds directly increase FXR target gene expression. (A-E) Huh7 cells were treated for 6 hours with either DMSO, 10 μM CDCA or 10 μM compound and were analysed for gene expression of FXR target genes *OSTα*, *OSTβ*, *SHP* and *FGF19*. This experiment (n=4) was performed in duplicate. Data is given as means ± SD. Significance is measured relative to DMSO controls. *$p < .05$ (One-way ANOVA; post hoc: Dunnett's multiple comparison).

Identification of FDA-approved drugs targeting the Farnesoid X receptor

Sandra M.W. van de Wiel[1,2], Ingrid T.G.W. Bijsmans[3], Saskia W.C. van Mil[1,3] and Stan F.J. van de Graaf[1,2]

[1]Tytgat Institute for Liver and Intestinal Research & [2]Department of Gastroenterology and Hepatology, Amsterdam Gastroenterology and Metabolism, Amsterdam UMC, University of Amsterdam, Amsterdam, the Netherlands. [3]Center for Molecular Medicine, UMC Utrecht, Utrecht University, Utrecht, The Netherlands.

Supplementary table 1

Primer list for RT-qPCR analysis

Gene	Forward primer (5'-3')	Reverse primer (5'-3')
HPRT	TGACCTTGATTTATTTTGCATACC	CGAGCAAGACGTTCAGTCCT
36B4	TCATCAACGGTACAAACGA	GCCTTGACCTTTTCAGCAAG
SLC51A (OSTα)	AGATAACGCTGACCCTGGTG	AATTTGGCTCCCATGTTCTG
SLC51B (OSTβ)	GCAGCTGTGGTGGTCATTAT	TAGGCTGTTGTGATCCTTGG
ABCC2 (MRP2)	ACAGAGGCTGGTGGCAACC	ACCATTACCTTGTCACTGTCCATGA
NR0B2 (SHP)	CGCCCTATCATTGGAGATGT	TGTCTATACAGGCTTGCCCC
FGF19	TTTCTCATCACTTCCCCAGG	AGGCTTCCCCTACTCCTGAA
G6Pase	CCTTGCTGCTCATTTTCCTC	GGCTGGCATTATAGATGCTGT
CYP7A1	AATTCCATACCTGGGCTGTG	AGGCAGCGGTCTTTGAGTTA
